# A three-phase epidemiological fluctuation framework of extrapulmonary tuberculosis in Fuyang, China: a retrospective study

**DOI:** 10.3389/fpubh.2026.1914382

**Published:** 2026-09-09

**Authors:** Tuantuan Li, Xiaowu Wang, Mei Li, Yunyun Ding, Yong Gao, Yan Liu

**Affiliations:** 1 Department of Clinical Laboratory, The Second People’s Hospital of Fuyang City, Fuyang, Anhui, China; 2 The Third People’s Hospital of Fuyang City, Fuyang, Anhui, China

**Keywords:** diagnosis, drug resistance, epidemiological transition, epidemiology, extrapulmonary tuberculosis, GeneXpert MTB/RIF ultra, IGRA, *Mycobacterium tuberculosis* culture

## Abstract

**Objective:**

This study characterized eight-year epidemiological and clinical features, diagnostic performance, and drug resistance of EPTB in Fuyang, China, and proposed a framework to interpret its dynamic burden.

**Methods:**

We conducted a retrospective study of EPTB patients at Fuyang Second People’s Hospital from January 2018 to December 2025. Demographic, clinical, and laboratory data were extracted from medical records. Descriptive statistics and chi-square tests were used to analyze epidemiological characteristics. Diagnostic yields of IGRA, GeneXpert MTB/RIF Ultra, and culture were compared based on positivity rates. Drug susceptibility testing (DST) was performed on culture-positive isolates. MDR was defined as resistance to at least isoniazid and rifampicin.

**Results:**

EPTB accounted for 28.46% (2,281/8015) of all tuberculosis (TB) cases. The majority of patients were aged (≥50 years, 52.78%), male (70.45%), and farmers (70.50%). Temporal analysis revealed a distinctive fluctuation pattern, with EPTB cases predominantly concentrated in 2019–2021 and 2024, which we interpret as reflecting a three-phase fluctuation framework. The most prevalent subtypes of EPTB were pleural TB (60.50%), osteoarticular TB (12.98%), lymphatic TB (6.97%), central nervous system (4.65%), and gastrointestinal TB (3.24%). IGRA showed the highest positivity rate (88.04%), significantly higher than GeneXpert MTB/RIF Ultra (35.60%) and culture (14.53%) (*p* < 0.001). Clinical characteristics differed across the five most common EPTB subtypes. Among the 54 isolates that underwent DST, resistance rates were highest for isoniazid (14.81%) and ethambutol (11.11%). Overall, 83.33% of these tested isolates were fully susceptible, while 9.26% were multidrugresistant (MDR), accounting for 55.60% of all resistant cases within this tested subset.

**Conclusion:**

EPTB constitutes a significant burden, particularly among aged, male, and farming populations. Pleural TB is the most common form, and IGRA offers superior detection yield. Notably, the 2018–2025 EPTB proportion fluctuations fit a three-phase descriptive framework (diagnosis-driven ascendancy, pandemic disruption, re-equilibration), inspired by Omran’s theory as a heuristic analogy, with breakpoints based on annual trends rather than statistically derived. Although overall drug resistance was low among the tested isolates, the presence of MDR cases in this selected sample underscores the importance of routine DST; however, larger unselected studies are warranted to validate the true population-level MDR burden.

## Introduction

Tuberculosis (TB) remains a leading cause of death from a single infectious agent worldwide, representing a persistent global public health challenge. According to the World Health Organization (WHO), the global TB incidence rate in 2024 was 131 new cases per 100,000 population, with an estimated 10.7 million people developing the disease and 1.23 million deaths (case fatality rate: 11.5%), and with China ranking fourth globally and accounting for 6.5% of the world’s total cases among 30 highburden countries (1). TB typically affects the lungs (Pulmonary TB, PTB) but can also involve other sites (Extrapulmonary Tuberculosis, EPTB), including the pleura, lymph nodes, bones, joints, genitourinary tract, and central nervous system (2). EPTB poses distinct diagnostic challenges due to its nonspecific clinical presentations and the frequently paucibacillary nature of extrapulmonary specimens, often resulting in delayed diagnosis and increased morbidity (3). Extrapulmonary tuberculosis (EPTB) constitutes a substantial proportion of the global TB burden, accounting for approximately 15–20% of all TB cases worldwide, with notified cases in 2024 representing 16% of all newly diagnosed TB cases globally (4). However, the true global burden of EPTB is likely underestimated due to diagnostic challenges and underreporting, with reported proportions varying widely across different countries and regions (5). EPTB can affect virtually any organ system (4). The diagnosis of EPTB is further complicated by its nonspecific clinical presentations and the frequently paucibacillary nature of extrapulmonary specimens (6). Patients with EPTB experience considerable diagnostic delays, with studies reporting median health system delays of up to 4 weeks and total treatment delays exceeding 10 weeks (7). A study involving 31 medical institutions randomly selected from 21 provinces in China revealed that, among 6,843 clinically diagnosed or laboratoryconfirmed tuberculosis patients, 1,681 (24.6%) were identified as having extrapulmonary tuberculosis (8).

Despite substantial progress in TB control over the past two decades, China continues to face significant challenges, particularly regarding the diagnosis and management of EPTB. However, its epidemiological characteristics remain less characterized than those of PTB, particularly at the subnational level. A previous study documented 211,892 pulmonary TB cases in Anhui Province, eastern China, between 2013 and 2018, corresponding to an average annual reported incidence rate of 57.7 per 100,000 population (9). The Fuyang area, a populous prefecturelevel city in northwestern Anhui with over 10 million residents, carries a substantial TB burden (10). As the secondlargest city in the province and a major population hub, Fuyang serves as a key sentinel site for TB surveillance. The authors’ affiliated institution, Fuyang Second People’s Hospital, functions as the designated regional referral center for TB, ensuring the availability of representative and centralized case data offering a unique opportunity to examine realworld EPTB management within a highpriority epidemiological context. Nevertheless, comprehensive, longterm data on the clinical spectrum and diagnostic modalities of EPTB in this specific setting remain scarce.

Existing diagnostic modalities for EPTB, including interferongamma release assays (IGRA), nucleic acid amplification tests (NAATs) like GeneXpert MTB/RIF Ultra, and conventional mycobacterial culture, have variable performance depending on the disease site and patient population, with sensitivities ranging from 75% in cerebrospinal fluid to 100% in pleural specimens for GeneXpert Ultra (11). Nevertheless, their real-world performance across different EPTB anatomical sites in routine Chinese clinical settings requires further evaluation, as current evidence from China remains limited and primarily derived from single-center or regional studies. Previous studies have identified several demographic and clinical factors associated with EPTB, including age >60 years, female gender, comorbidities, and severe symptoms (12, 13). However, these findings are largely based on limited hospital-based data and require further validation in different epidemiological settings.

The epidemiological transition theory, originally proposed by Omran in 1971, describes a three-phase framework of how human societies shift from infectious diseases to chronic noncommunicable diseases as the predominant cause of morbidity and mortality, and has become a classic theoretical framework in demography and public health (14). This theory has been widely applied to explain structural changes in disease burden across different countries and regions. In recent years, researchers have begun to apply this framework to analyze the reemergence of infectious diseases and shifts in the burden of specific diseases. The present study draws on this classic theory as an analytical framework and applies it creatively to extrapulmonary tuberculosis as a specific disease, in order to explain the phased changes in the proportional composition of EPTB in the Fuyang region.

To address these gaps, we conducted an eight-year retrospective cohort study (2018–2025) at Fuyang Second People’s Hospital, a 1,200-bed tertiary-care infectious disease referral center in Anhui Province, China. The specific objectives of this study were: (1) to delineate the epidemiological profile and clinical spectrum of EPTB in this high-burden, resource-limited setting; (2) to quantify the detection yields (positivity rates) of the diagnostic assays employed for EPTB across diverse anatomical sites, while acknowledging that the absence of a gold-standard reference precludes formal detection yield assessment; (3) to describe the predominant EPTB phenotypes and their corresponding drug resistance patterns; and (4) to propose, using Omran’s epidemiological transition theory as an analogical and hypothesis-generating framework (14), a descriptive “three-phase fluctuation” model for EPTB comprising diagnosis-driven ascendancy (2018–2020), pandemic-associated disruption (2021–2022), and post-pandemic re-equilibration (2023–2025) as a tentative interpretive lens for the dynamic proportional changes observed over the study period. We emphasize that the phase breakpoints are descriptive categorizations based on annual trend observations and are not statistically derived; this framework is intended to generate testable hypotheses for future validation rather than to assert a definitive transitional paradigm. The detailed drug resistance patterns and sitespecific diagnostic yields provide a robust foundation for optimizing EPTB care in this region.

## Materials and methods

**Figure 1:**
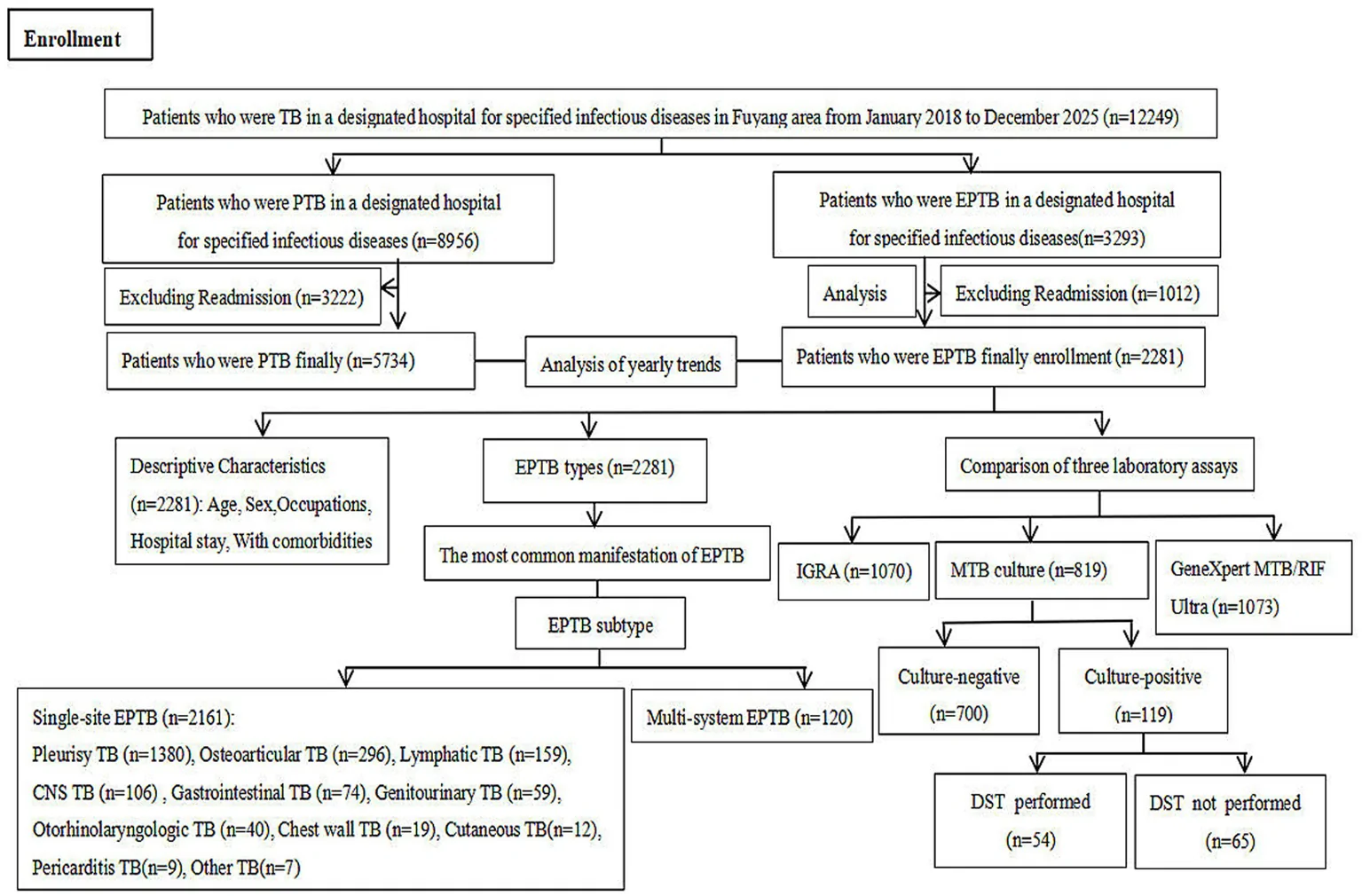
Flowchart illustrating patient enrollment. TB, tuberculosis; *n*, number; PTB, pulmonary tuberculosis; EPTB, extrapulmonary tuberculosis; IGRA, Interferon-gamma release assay; MTB, *Mycobacterium tuberculosis*; CNS TB, Central nervous system tuberculosis; DST, drug susceptibility testing.

This was a retrospective study conducted over a defined period from January 1, 2018, to December 31, 2025, and all eligible patients with tuberculosis during this timeframe were initially included (*n* = 12,249). After excluding readmissions and classifying patients into pulmonary tuberculosis (PTB) and extrapulmonary tuberculosis (EPTB), a total of 5,734 PTB patients and 2,281 EPTB patients were finally enrolled for analysis. All consecutive eligible patients during the study period were included to maximize the available data. The patient enrollment process, including the detailed inclusion and exclusion criteria, is summarized in Figure 1.

### Data collection

Demographic, clinical, and laboratory data were extracted from medical records using a standardized data collection form. Collected variables included:

*Demographics*: age, sex, occupations (farmers/others).
*Clinical features*: types of TB involvement (EPTB only vs. EPTB plus PTB), EPTB anatomical sites, hospital length of stay.
*Comorbidities*: pulmonary TB, pulmonary infection, hypertension (HTN), diabetes mellitus (DM), coronary artery disease (CAD), chronic obstructive pulmonary disease (COPD), acquired immunodeficiency syndrome (AIDS), chronic kidney disease (CKD), and communityacquired pneumonia (CAP).
*Diagnostic test results*: interferongamma release assay (IGRA), Gene Xpert MTB/RIF Ultra, and *Mycobacterium tuberculosis* (MTB) culture.

### Definitions

Extrapulmonary tuberculosis (EPTB): Tuberculosis involving organs other than the lungs, confirmed by clinical, radiological, microbiological, or histopathological criteria.

Pulmonary tuberculosis (PTB): Diagnosed based on sputum smear, culture, GeneXpert MTB/RIF Ultra, or radiographic evidence consistent with active pulmonary TB.

EPTB plus PTB: Concurrent pulmonary and extrapulmonary involvement.

Singlesite vs. Multisystem EPTB: Classified by the number of noncontiguous anatomical sites involved.

### Interferongamma release assay

Performed using a commercial kit. Venous blood (3–5 mL) was collected into lithium heparin tubes. Peripheral blood mononuclear cells (PBMCs) were isolated by densitygradient centrifugation, then stimulated with *M. tuberculosis* antigens (ESAT6, CFP10), a phytohaemagglutinin positive control, and a mediumonly negative control. After 16–24 h of incubation (37 °C, 5% CO₂), supernatant IFNγ levels were measured by ELISA. A positive result required the antigenstimulated IFNγ value to be above the cutoff and at least double the negative control value. Indeterminate results (positive control failure or high negative control background) led to test repetition or further clinical evaluation.

### GeneXpert MTB/RIF ultra

Performed according to the manufacturer’s instructions. Specimen reagent (1.4 mL) was mixed with 0.7 mL of aspirate or homogenized tissue. Results were categorized as invalid (no internal control detected), not detected, or detected (with semiquantitative levels: trace, very low, low, medium, high). Rifampicin resistance was reported as detected, not detected, or indeterminate. Personnel conducting Xpert were blinded to all other results.

### *Mycobacterium tuberculosis* culture

Respiratory specimens were cultured using the automated BACTEC™ MGIT™ 960 system (Becton, Dickinson and Company, United States) and a modified Roche culture method. Isolates were preliminarily identified by susceptibility to Pnitrobenzoic acid (PNB) and pyridine2carboxylic acid hydrazide (TCH).

### Drug susceptibility testing

During the study period, DST was not routinely performed for all culture-positive patients. As a result, only 54 of the 119 culture-positive isolates underwent DST and were included in this analysis. The remaining 65 culture-positive patients did not undergo DST and were therefore excluded from the drug resistance analysis. Accordingly, the drug resistance data presented herein reflect findings from this subset and may not be generalizable to the entire culture-positive cohort.

Performed using the *Mycobacterium tuberculosis* Drug Susceptibility Testing Kit (broth microdilution method for minimal inhibitory concentration, MIC; Zhengzhou Antu Biological Engineering Co., Ltd., China) on the BACTEC MGIT 960 system. The kit tests 13 antituberculosis drugs: rifampicin (R, RFP), isoniazid (H, INH), streptomycin (S, SM), ethambutol (E, EMB), rifabutin (RFB), ofloxacin (OFX), levofloxacin (LFX), moxifloxacin (MFX), amikacin (AM), kanamycin (KM), capreomycin (CM), ethionamide (ETO), and paraaminosalicylic acid (PAS). Each batch included the reference strain *M. tuberculosis* H37Ra (ATCC 25177) as a susceptible control. Resistance was defined using WHOrecommended critical concentrations: H (0.2 μg/mL), R (1.0 μg/mL), E (5.0 μg/mL), S (2.0 μg/mL), Rfb (0.5 μg/mL), Ofx (2.0 μg/mL), Lfx (2.0 μg/mL), Mfx (0.5 μg/mL), Am (1.0 μg/mL), Km (5.0 μg/mL), Cm (2.0 μg/mL), Eto (2.5 μg/mL), PAS (2 μg/mL). No visible colony growth indicated susceptible (S); visible growth indicated resistant (R). Line probe assay (LPA) was not used.

### Antituberculosis drugs

The DST panel evaluated in this study comprised the following 13 anti-TB agents, categorized for analysis as first-line agents (rifampicin [R], isoniazid [H], and ethambutol [E]), injectable second-line agents (streptomycin [S], amikacin [Am], kanamycin [Km], and capreomycin [Cm]), fluoroquinolones (ofloxacin [Ofx], levofloxacin [Lfx], and moxifloxacin [Mfx]), and other oral second-line drugs (rifabutin [Rfb], ethionamide [Eto], and para-aminosalicylic acid [PAS]). Importantly, pyrazinamide (PZA), although conventionally classified as a first-line agent, was not included in the testing panel and was therefore excluded from all resistance analyses in the present study.

### Statistical analysis

Statistical analyses were performed using SPSS version 26.0 (IBM Corp., United States). Categorical variables were presented as frequencies and percentages, and compared using the chisquare test or Fisher’s exact test when expected cell counts were <5. Continuous variables were expressed as mean ± standard deviation (SD) or median with interquartile range (IQR), and compared using Student’s ttest or the MannWhitney U test, as appropriate. A pvalue <0.05 was considered statistically significant. Phase divisions (2018–2020, 2021–2022, 2023–2025) were defined descriptively based on annual trend inspection, without formal statistical breakpoint analysis.

## Results

### The overall epidemiology of extrapulmonary tuberculosis

**Figure 2:**
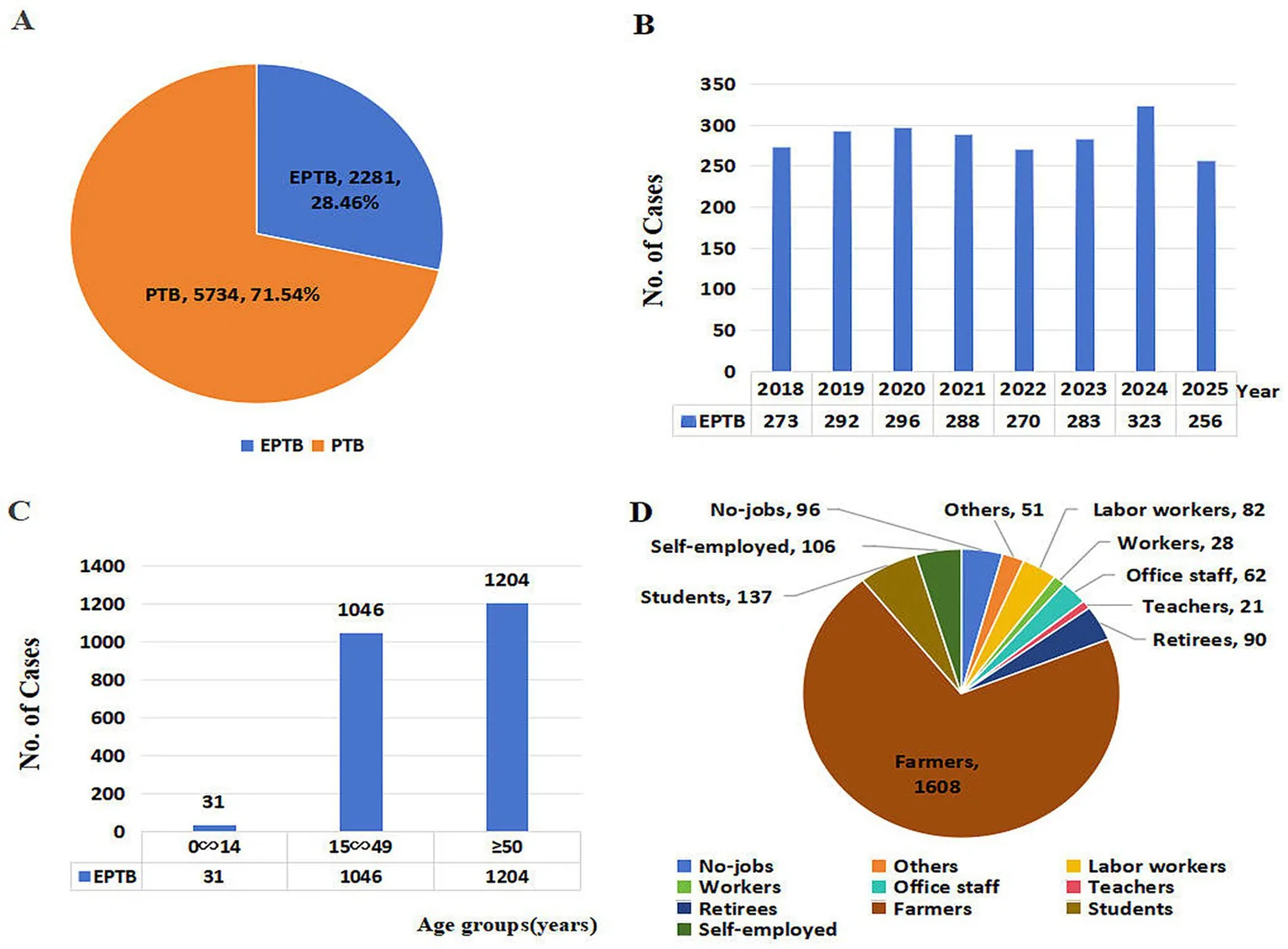
Distribution of extrapulmonary tuberculosis from 2018 to 2025. **(A)** Distribution of pulmonary tuberculosis, extrapulmonary tuberculosis, and tuberculosis patients; **(B)** Annual distribution of extrapulmonary tuberculosis patients; **(C)** Age distribution of extrapulmonary tuberculosis patients; **(D)** Occupational distribution of extrapulmonary tuberculosis patients. PTB, pulmonary tuberculosis; EPTB, extrapulmonary tuberculosis.

During the period from 2018 to 2025, a total of 8,015 TB cases were diagnosed at Fuyang Second People’s Hospital (Figure 2A). Among these, 5,734 were PTB cases and 2,281 were EPTB cases (including those with or without concurrent PTB), accounting for 28.46% (2,281/8015) of all TB cases.

From 2018 to 2025, the number of EPTB cases fluctuated (Figure 2B). Specifically, the number of cases increased from 273 in 2018 to 292 in 2019, reaching a minor peak of 296 in 2020. This was followed by a consecutive decline in 2021 (288 cases) and 2022 (270 cases), with a slight rebound to 283 cases in 2023. In 2024, the number of cases increased markedly, reaching the highest peak during the observation period (323 cases), followed by a sharp decline to the lowest level in 2025 (256 cases). This temporal pattern exhibits distinct phases of ascent, decline, and rebound that merit further analytical interpretation (see Discussion).

Among patients with EPTB, the age ranged from 4 to 92 years. The distribution of cases across the 0–14 years group (31 cases), the 15–49 years group (1,046 cases), and the ≥50 years group (1,204 cases) (Figure 2C). In terms of occupational distribution (Figure 2D), farmers were the predominant group among extrapulmonary tuberculosis patients, accounting for 70.50% (1,608/2281). Students and the unemployed represented 6.01% (137/2281) and 4.65% (106/2281) of cases, respectively.

### Baseline characteristics of the study population

A total of 2,281 patients with EPTB were included (Table 1). The mean age was 50.35 ± 21.04 years (range: 4–92 years), with patients aged ≥50 years accounting for 52.78% (1,204/2281) of the cohort. Males comprised 70.45% (1,607/2281) of the study population. Farmers represented the predominant occupational group, accounting for 70.50% (1,608/2281) of cases. The mean hospital stay was 14.57 ± 11.96 days. Regarding comorbidities, pulmonary infection was the most common (73.74%, 1682/2281), followed by hepatobiliary diseases (24.68%, 563/2281), malnutrition (including anemia and hypoproteinemia) (22.53%, 514/2281), gastrointestinal diseases (20.34%, 464/2281), hypertension (17.10%, 390/2281), and diabetes mellitus (9.30%, 212/2281). Additionally, 54.45% (1,242/2281) of patients had concurrent pulmonary tuberculosis.

**Table 1:** Baseline characteristics of patients with extrapulmonary tuberculosis [*n* (%)].

Baseline characteristics	Patients (*n* = 2,281)
Age, years, Mean (SD)	50.35 (21.04)
Age group (years), *n* (%)	
0∽14	31 (1.36)
15∽49	1,046 (45.86)
≥50	1,204 (52.78)
Male, *n* (%)	1,607 (70.45)
Farmers, *n* (%)	1,608 (70.50)
Hospital stay, days, Mean (SD)	14.57 (11.96)
Underlining diseases, *n* (%)	
DM	212 (9.30)
HTN	390 (17.10)
Pulmonary infection	1,682 (73.74)
CAD	105 (4.60)
Malnutrition (anemia, hypoproteinemia)	514 (22.53)
Renal insufficiency	222 (9.73)
Hepatobiliary diseases	563 (24.68)
AIDS	64 (2.81)
Gastrointestinal diseases	464 (20.34)
thyroid diseases	35 (1.53)
Mental illness	11 (0.48)
Tumor	40 (1.75)
PTB	1,242 (54.45)

SD, standard deviation; DM, diabetes mellitus; HTN, hypertension; CAD, coronary artery disease; AIDS, acquired immunodeficiency syndrome; PTB, pulmonary tuberculosis.

### Spectrum and distribution of extrapulmonary tuberculosis types

**Figure 3:**
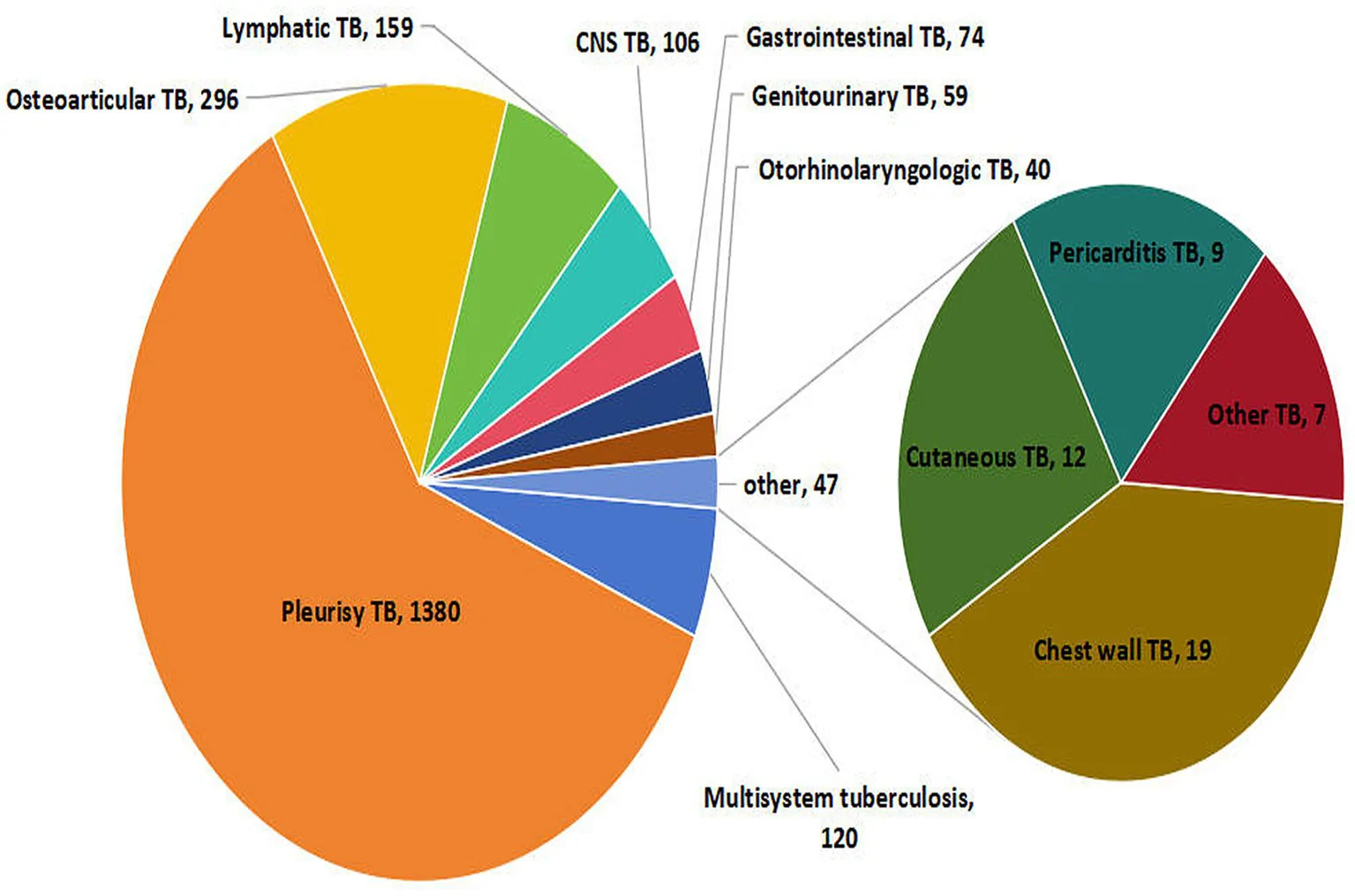
Composition of extrapulmonary tuberculosis types. EPTB, extrapulmonary tuberculosis; CNS, central nervous system.

Among 2,281 EPTB patients, singlesite disease predominated (94.74%, 2161/2281), while multisystem involvement was less frequent (5.26%, 120/2281). Pleural TB was the most common subtype (60.50%, 1380/2281), followed by osteoarticular TB (12.98%, 296/2281) and lymphatic TB (6.97%, 159/2281). Central nervous system (CNS), gastrointestinal TB, and genitourinary TB accounted for 4.65% (106/2281), 3.24% (74/2281), and 2.59% (59/2281) of cases, respectively. Rarer forms (e.g., otorhinolaryngologic, chest wall, cutaneous, pericarditis, other) each comprised <2.00% of the cohort (Figure 3).

### Trend analysis of extrapulmonary tuberculosis from 2018 to 2025

**Figure 4:**
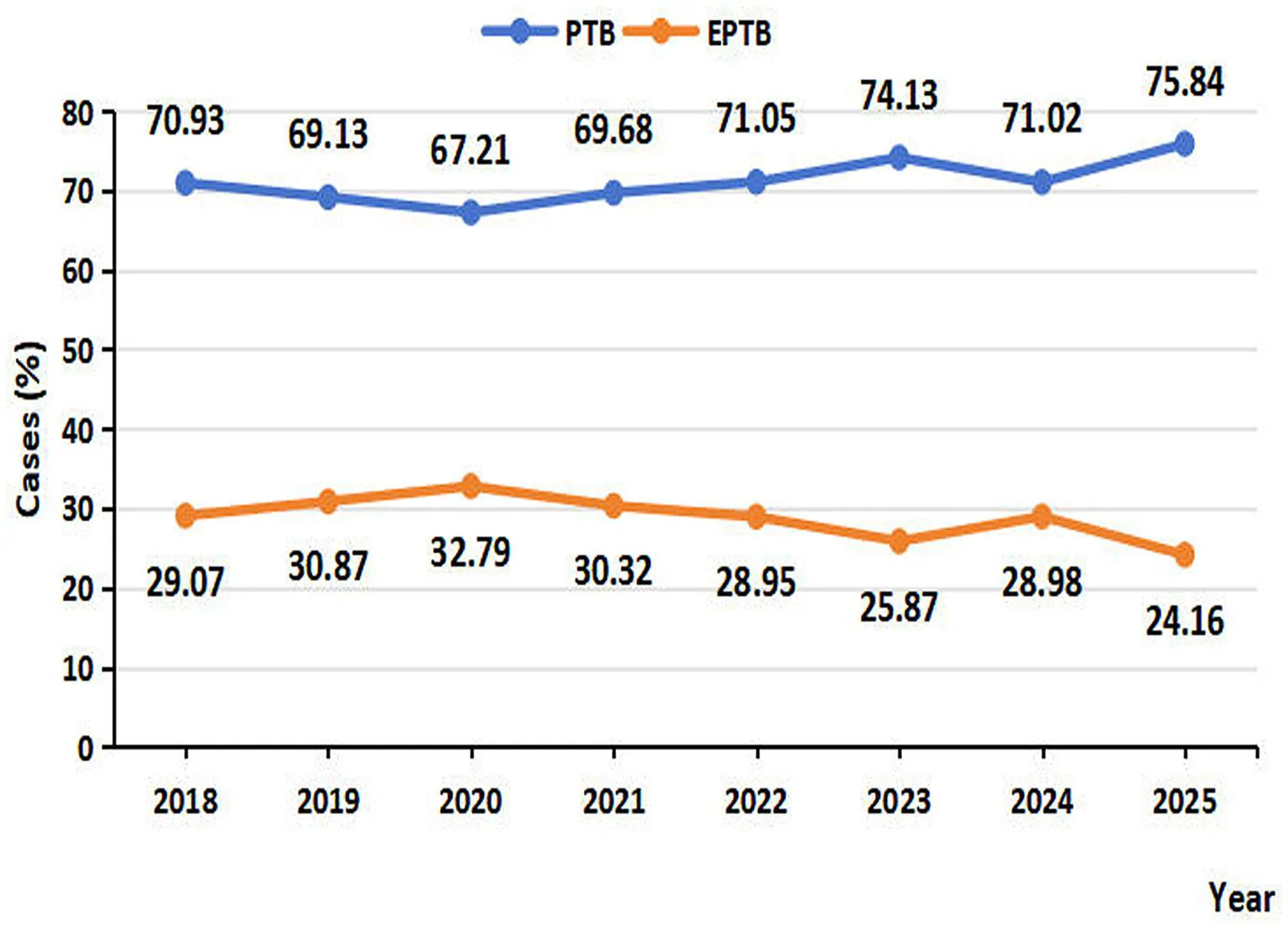
Relative incidence of extrapulmonary tuberculosis and pulmonary tuberculosis from 2018 to 2025. PTB, pulmonary tuberculosis; EPTB, extrapulmonary tuberculosis.

Over the study period from 2018 to 2025, the annual proportion of EPTB among all tuberculosis cases fluctuated, ranging from 24.16 to 32.79% (Figure 4). The highest EPTB proportion was observed in 2020 (32.79%), followed by a gradual decline to the lowest level in 2025 (24.16%). Conversely, the proportion of pulmonary tuberculosis (PTB) showed an inverse trend, increasing from 67.21% in 2020 to 75.84% in 2025. This inverse relationship between the proportional trends of EPTB and PTB over time is noteworthy and will be explored in relation to healthcare system factors in the Discussion.

### Relative incidence of different anatomical sites of extrapulmonary tuberculosis infection from 2018 to 2025

**Figure 5:**
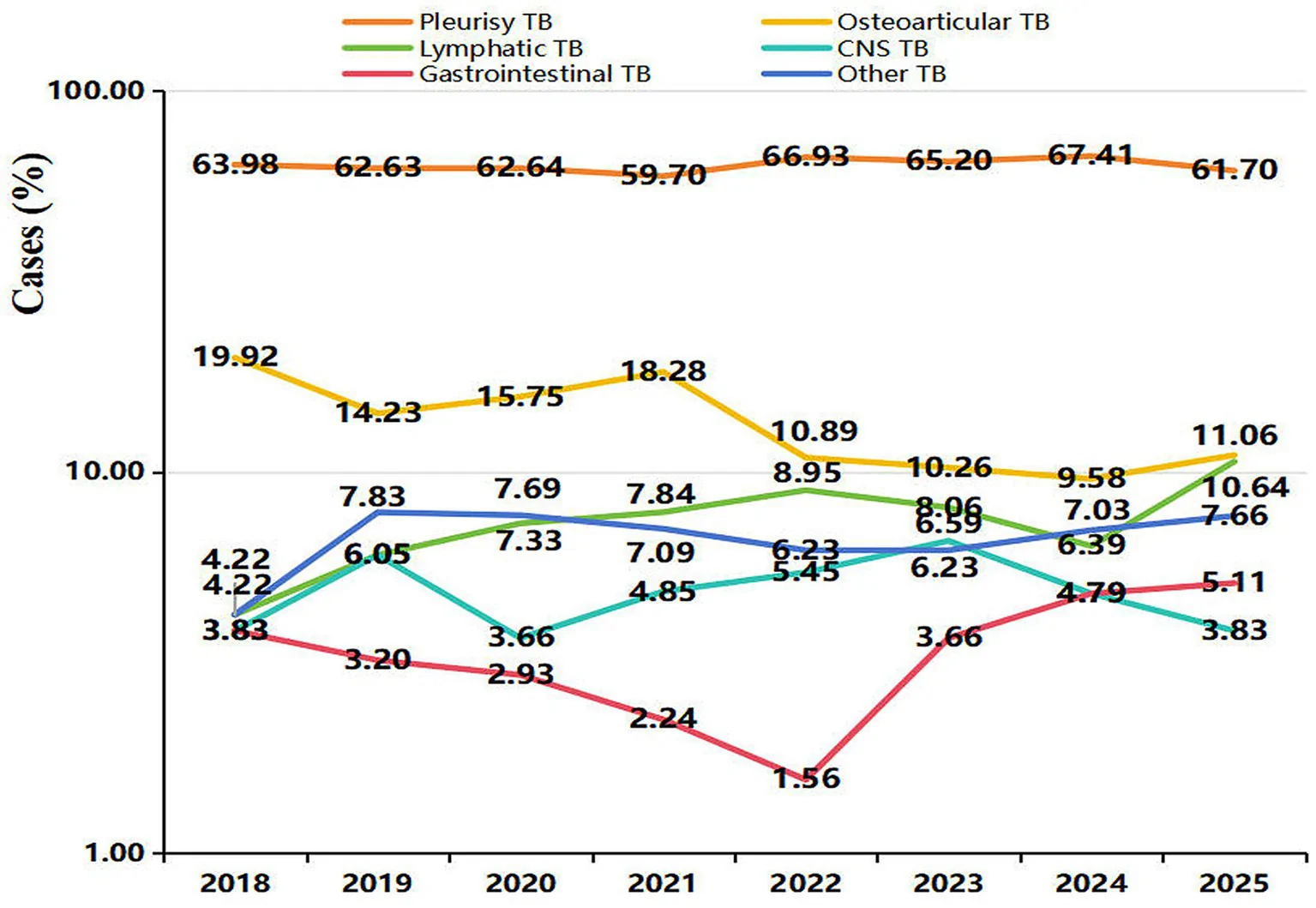
Trends in the anatomical site of TB infection from 2018 to 2025. TB, tuberculosis; CNS, Central Nervous System.

We further characterized trends in the anatomical site of TB infection from 2018 to 2025 (Figure 5). Pleural TB consistently remained the predominant form, with proportions fluctuating between 59.70 and 67.41%; it peaked in 2022 (66.93%) and 2024 (67.41%) before decreasing to 61.70% in 2025. Osteoarticular TB exhibited a general declining trend, dropping from 19.92% in 2018 to 11.06% in 2025, with its lowest point recorded in 2024 (9.58%). Conversely, Lymphatic TB demonstrated a marked upward trend, increasing from 4.22% in 2018 to 10.64% in 2025. CNS TB showed moderate fluctuations, ranging from 3.66 to 6.59% during the study period. Gastrointestinal TB, after declining to its lowest point of 1.56% in 2022, subsequently rebounded to 5.11% by 2025. Other TB (including otorhinolaryngologic, chest wall, cutaneous, pericardial, and other rare forms) proportions increased from 4.22% in 2018 to 7.83% in 2019, remained relatively stable at approximately 6−7% over the following years (with a low of 6.23% in 2022−2023), and eventually reached 7.66% by 2025. The contrasting trajectories between osteoarticular TB (declining) and lymphatic TB (rising) highlight subtypespecific heterogeneity in the evolving EPTB case mix.

### Clinical characteristics of the five most common singlesystem extrapulmonary tuberculosis subtypes

Table 2 summarizes the clinical characteristics of the five most common single-system EPTB subtypes. Among these subtypes, the highest proportions for the key variables were distributed as follows: male predominance in pleural TB (76.67%), patients aged ≥50 years in osteoarticular TB (77.36%), farmers in osteoarticular TB (79.05%), concurrent pulmonary TB in CNS TB (89.62%), hepatobiliary diseases in CNS TB (44.34%), malnutrition in gastrointestinal TB (40.54%), HIV/AIDS in CNS TB (13.21%), and diabetes in osteoarticular TB (13.18%). Moreover, IGRA and Xpert MTB/RIF Ultra positivity rates were both highest in lymphatic TB (96.36 and 80.00%, respectively), while MTB culture positivity was highest in osteoarticular TB (36.25%).

**Table 2:** Clinical characteristics of patients with the five most common single-system types of extrapulmonary tuberculosis.

Variable	Pleurisy TB (*n* = 1,380)	Osteoarticular TB (*n* = 296)	Lymphatic TB (*n* = 159)	CNS TB (*n* = 106)	Gastrointestinal TB (*n* = 74)
Age range, *n* (%)					
0∽14	16 (1.16)	5 (1.69)	2 (1.26)	2 (1.89)	1 (1.35)
15∽49	676 (48.99)	62 (20.95)	105 (66.04)	38 (35.85)	36 (48.65)
≥50	688 (49.86)	229 (77.36)	52 (32.70)	66 (62.26)	37 (50.00)
Male, *n* (%)	1,058 (76.67)	175 (59.12)	77 (48.43)	69 (65.09)	51 (68.92)
Farmers, *n* (%)	942 (68.26)	234 (79.05)	98 (61.64)	81 (76.42)	56 (75.68)
Tumor, *n* (%)	19 (1.38)	6 (2.03)	2 (1.26)	2 (1.89)	3 (4.05)
Pulmonary infection, *n* (%)	600 (43.48)	120(40.54)	109 (68.55)	95 (89.62)	56 (75.68)
Mental illness, *n* (%)	7 (0.51)	1 (0.34)	0 (0.00)	1(0.94)	1 (1.35)
Thyroid diseases, *n* (%)	20 (1.45)	2 (0.68)	6 (3.77)	2 (1.89)	1 (1.35)
Gastrointestinal diseases, *n* (%)	279 (20.22)	35 (11.82)	29 (18.24)	36 (33.96)	20 (27.03)
AIDS, *n* (%)	24 (1.74)	5 (1.69)	4 (2.52)	14 (13.21)	7 (9.46)
Hepatobiliary diseases, *n* (%)	329 (23.84)	45 (15.20)	42 (26.42)	47 (44.34)	26 (35.14)
Renal insufficiency, *n* (%)	107 (7.75)	24 (8.11)	8 (5.03)	6 (5.66)	18 (24.32)
Malnutrition (anemia, hypoproteinemia), *n* (%)	311 (22.54)	52 (17.57)	13 (8.18)	34 (32.08)	30 (40.54)
CAD, *n* (%)	71 (5.14)	16 (5.41)	4 (2.52)	4 (3.77)	2 (2.70)
DM, *n* (%)	133 (9.64)	39 (13.18)	9 (5.66)	12 (11.32)	2 (2.70)
HTN, *n* (%)	229 (16.59)	77 (26.01)	18 (11.32)	19 (17.92)	8 (10.81)

TB, tuberculosis; CNS, Central nervous system; AIDS, acquired immunodeficiency syndrome; CAD, coronary artery disease; DM, diabetes mellitus; HTN, hypertension.

### Diagnostic yield of extrapulmonary tuberculosis assays

Table 3 summarizes the diagnostic yields of IGRA (on peripheral blood), GeneXpert MTB/RIF Ultra, and MTB culture for EPTB, both overall and stratified by specimen type (for Ultra and culture). Overall, IGRA showed the highest positivity rate (88.04%, 942/1070), followed by GeneXpert MTB/RIF Ultra (35.60%, 382/1073), while MTB culture had the lowest yield (14.53%, 119/819); the difference among the three assays was statistically significant (*χ*^2^ = 1119.19, *p* < 0.001). When stratified by specimen type, substantial variation in detection yields was observed. For pleural fluid (pleural TB), IGRA positivity (from blood) remained high at 90.08% (581/645), whereas GeneXpert Ultra and culture yielded moderate (25.91%, 185/714) and low (10.95%, 60/548) positivity rates, respectively. In lymph node tissue (lymphatic TB), all three assays yielded relatively high positivity rates, with IGRA (blood) at 96.36% (53/55), GeneXpert Ultra at 80.00% (52/65), and culture at 20.45% (9/44). For pus/synovial fluid (osteoarticular TB), GeneXpert Ultra and culture achieved positivity rates of 51.02% (50/98) and 36.25% (29/80), respectively, while IGRA (blood) was positive in 75.43% (132/175). In cerebrospinal fluid (CSF) from CNS TB cases, culture yield was extremely low (2.44%, 1/41), and GeneXpert Ultra positivity was 29.09% (16/55); IGRA on blood showed 82.69% (43/52) positivity but cannot distinguish active from latent infection. For gastrointestinal TB (specimens primarily from ascitic fluid or tissue biopsies), IGRA (blood) positivity was 82.76% (24/29), followed by GeneXpert Ultra (21.74%, 5/23) and culture (6.67%, 1/15). These findings highlight the marked site- and specimen-dependent variability in diagnostic test yields for EPTB, with the caveat that IGRA results reflect a systemic immune response rather than local pathogen detection.

**Table 3:** Diagnostic yield of IGRA (performed on peripheral blood), GeneXpert MTB/RIF Ultra (on specified specimens), and MTB culture (on specified specimens) for extrapulmonary tuberculosis: Overall and stratified by specimen type for Xpert Ultra and culture.

Specimen type *for Xpert Ultra and culture* (Corresponding EPTB subtype)	No. of specimens (IGRA / Xpert Ultra / Culture)	IGRA positivity, n/N (%)	Xpert Ultra positivity, n/N (%)	MTB culture positivity, n/N (%)
Overall (all specimens)	1,070/1,073/819	942/1070 (88.04)	382/1073 (35.60)	119/819 (14.53)
By specimen type				
Pleural fluid (pleural TB)	645/714 / 548	581/645 (90.08)	185/714 (25.91)	60/548 (10.95)
Lymph node tissue (lymphatic TB)	55/65 / 44	53/55 (96.36)	52/65 (80.00)	9/44 (20.45)
Pus / synovial fluid (osteoarticular TB)	175/98 / 80	132/175 (75.43)	50/98 (51.02)	29/80 (36.25)
Cerebrospinal fluid (CSF) (CNS TB)	52/55 / 41	43/52 (82.69)	16/55 (29.09)	1/41 (2.44)
–* (gastrointestinal TB)	29/23 / 15	24/29 (82.76)	5/23 (21.74)	1/15 (6.67)

EPTB, extrapulmonary tuberculosis; TB, tuberculosis; IGRA, interferon-gamma release assay; MTB, *Mycobacterium tuberculosis*; CNS, central nervous system. –*, Gastrointestinal TB specimens for Xpert Ultra and culture were primarily from ascitic fluid or tissue biopsies; the small sample size precluded separate specimen-type stratification. Statistical comparison across assays (overall): *χ*^2^ = 1119.19, *p* < 0.001.

### Distribution of specimen sources among 119 culturepositive extrapulmonary tuberculosis patients

Among the 119 culturepositive EPTB patients, pleural fluid was the predominant specimen source, accounting for 59 cases (49.58%), suggesting that pleural effusion is the most common culturable form of EPTB (Table 4). This was followed by pus (19.33%) and biopsied tissue specimens (10.92%). These three specimen types together accounted for 79.83% of all positive cultures. Although specimens such as cerebrospinal fluid and ascitic fluid also yielded bacterial growth, the number of positive cases was relatively low.

**Table 4:** Distribution of specimen sources among 119 culture-positive extrapulmonary tuberculosis patients.

Specimen type	No. of isolates (*n*)	Proportion (%)
Pleural fluid	59	49.58
Pus	23	19.33
Tissue	13	10.92
Urine	8	6.72
Lymph node aspirate	7	5.89
Synovial fluid	6	5.04
Ascitic fluid	2	1.68
Cerebrospinal fluid	1	0.84
Total	119	100.0

### Drug resistance profiles of 54 clinical *Mycobacterium tuberculosis* isolates

Among the 119 culture-positive EPTB cases, 54 isolates underwent drug susceptibility testing against 13 antituberculosis drugs, including four first-line and 9 sec-line agents (Table 5). Among first-line drugs, the highest resistance rate was observed for INH (14.81%, 8/54), followed by EMB (11.11%, 6/54), RFP (9.26%, 5/54), and SM (5.56%, 3/54). For second-line drugs, resistance rates of 7.41% (4/54) were noted for MFX, PTO, and RFB; rates of 5.56% (3/54) were observed for LFX, CPM, OFX, AMK, and KM; and the lowest rate was for PAS (1.85%, 1/54). Sex-stratified analysis showed that INH resistance was 15.56% in males vs. 11.11% in females; EMB, RFP, and SM resistance occurred only in males, whereas CPM, AMK, and KM resistance were higher in females (11.11% vs. 4.44% each), but none of these sex differences reached statistical significance (all *p* > 0.05). Age-stratified comparisons revealed higher resistance rates in the 15–49 years group than in the ≥50 years group, notably for INH (23.08% vs. 7.14%), RFP (15.38% vs. 3.57%), SM (11.54% vs. 0%), and MFX (15.38% vs. 0%), yet all differences remained non-significant (*p* = 0.090–1.000); no patient was aged <15 years.

**Table 5:** Drug resistance profiles of 54 clinical *Mycobacterium tuberculosis* isolates stratified by sex and age.

Drug class	Drug name	Total (*N* = 54)	Male (*n* = 45)	Female (*n* = 9)	*p* value (Sex)	Age 15–49 years (*n* = 26)	Age ≥50 years (*n* = 28)	*p* value (Age)
First-line	INH	8 (14.81)	7 (15.56)	1 (11.11)	0.733	6 (23.08)	2 (7.14)	0.103
	EMB	6 (11.11)	6 (13.33)	0 (0.00)	0.571	4 (15.38)	2 (7.14)	0.299
	RFP	5 (9.26)	5 (11.11)	0 (0.00)	0.651	4 (15.38)	1 (3.57)	0.153
	SM	3 (5.56)	3 (6.67)	0 (0.00)	0.778	3 (11.54)	0 (0.00)	0.197
Second-line	MFX	4 (7.41)	4 (8.89)	0 (0.00)	0.700	4 (15.38)	0 (0.00)	0.090
	PTO	4 (7.41)	4 (8.89)	0 (0.00)	0.700	3 (11.54)	1 (3.57)	0.277
	RFB	4 (7.41)	4 (8.89)	0 (0.00)	0.700	3 (11.54)	1 (3.57)	0.277
	LFX	3 (5.56)	3 (6.67)	0 (0.00)	0.778	3 (11.54)	0 (0.00)	0.197
	CPM	3 (5.56)	2 (4.44)	1 (11.11)	0.427	3 (11.54)	0 (0.00)	0.197
	OFX	3 (5.56)	3 (6.67)	0 (0.00)	0.778	3 (11.54)	0 (0.00)	0.197
	AMK	3 (5.56)	2 (4.44)	1 (11.11)	0.427	3 (11.54)	0 (0.00)	0.197
	KM	3 (5.56)	2 (4.44)	1 (11.11)	0.427	3 (11.54)	0 (0.00)	0.197
	PAS	1 (1.85)	1 (2.22)	0 (0.00)	1.000	1 (3.85)	0 (0.00)	1.000

Data are presented as *n* (%). INH, isoniazid; EMB, ethambutol; RFP, rifampicin; SM, streptomycin; MFX, moxifloxacin; PTO, prothionamide; RFB, rifabutin; LFX, levofloxacin; CPM, capreomycin; OFX, ofloxacin; AMK, amikacin; KM, kanamycin; PAS, para-aminosalicylic acid. *p* values were calculated using two-sided Fisher’s exact test for 2 × 2 contingency tables. Intermediate (I) results were classified as resistant. No patient was aged 0–14 years in this cohort.

### Drug susceptibility patterns of 54 clinical *Mycobacterium tuberculosis* isolates

Of the 54 tested isolates, 45 (83.33%) were fully susceptible to all four first-line drugs, and 9 (16.67%) showed resistance to at least one drug (Table 6). Among the resistant isolates, 3 (5.56%) were mono-resistant (2 to H and 1 to E), 1 (1.85%) was poly-resistant (resistant to INH + EMB), and 5 (9.26%) were multidrug-resistant (MDR). The MDR isolates comprised 1 strain resistant to H + R + S, 2 strains resistant to H + R + E, and 2 strains resistant to H + R + E + S. Sex-stratified subgroup analyses revealed comparable full-susceptibility rates (82.22% vs. 88.89%, *p* = 0.570), with all 5 MDR isolates recovered from males (11.11% vs. 0%, *p* = 0.386). By age, the ≥50 years group showed a higher proportion of fully susceptible isolates (89.29% vs. 76.92%, *p* = 0.317), whereas MDR was more frequent in the 15–49 years group (15.38% vs. 3.57%, *p* = 0.153) (all *p* values by two-sided Fisher’s exact test; no patient aged <15 years).

**Table 6:** Drug susceptibility patterns of 54 clinical *M. tuberculosis* isolates stratified by sex and age (based on first-line drugs H, R, S, E).

Susceptibility/resistance pattern	Total (*N* = 54)	Male (*n* = 45)	Female (*n* = 9)	*p* value (Sex)	Age 15–49 years (*n* = 26)	Age ≥50 years (*n* = 28)	*p* value (Age)
Fully susceptible*	45 (83.33)	37 (82.22)	8 (88.89)	0.570	20 (76.92)	25 (89.29)	0.317
Any first-line resistance	9 (16.67)	8 (17.78)	1 (11.11)	0.570	6 (23.08)	3 (10.71)	0.317
Mono-resistant	3 (5.56)	2 (4.44)	1 (11.11)	0.427	1 (3.85)	2 (7.14)	0.528
H-resistant	2 (3.70)	1 (2.22)	1 (11.11)	0.308	1 (3.85)	1 (3.57)	1.000
R-resistant	0 (0.00)	0 (0.00)	0 (0.00)	—	0 (0.00)	0 (0.00)	—
S-resistant	0 (0.00)	0 (0.00)	0 (0.00)	—	0 (0.00)	0 (0.00)	—
E-resistant	1 (1.85)	1 (2.22)	0 (0.00)	1.000	0 (0.00)	1 (3.57)	1.000
Poly-resistant†	1 (1.85)	1 (2.22)	0 (0.00)	1.000	1 (3.85)	0 (0.00)	0.481
MDR (Total)	5 (9.26)	5 (11.11)	0 (0.00)	0.386	4 (15.38)	1 (3.57)	0.153
H + R	0 (0.00)	0 (0.00)	0 (0.00)	—	0 (0.00)	0 (0.00)	—
H + R + S	1 (1.85)	1 (2.22)	0 (0.00)	1.000	1 (3.85)	0 (0.00)	0.481
H + R + E	2 (3.70)	2 (4.44)	0 (0.00)	1.000	1 (3.85)	1 (3.57)	1.000
H + R + E + S	2 (3.70)	2 (4.44)	0 (0.00)	1.000	2 (7.69)	0 (0.00)	0.231

Data are presented as *n* (%). *p* values were calculated using two-sided Fisher’s exact test for 2 × 2 contingency tables; “—” indicates that the *p* value could not be estimated due to zero events in both subgroups. Intermediate (I) results were classified as resistant for phenotypic categorization. No patient was aged 0–14 years in this cohort. H, isoniazid; R, rifampicin; S, streptomycin; E, ethambutol; MDR, multidrug-resistant (resistant to at least H and R). *Fully susceptible indicates simultaneous susceptibility to H, R, S, and E. †Poly-resistant was defined as resistance to two or more first-line drugs without meeting the MDR criteria (in this cohort, the pattern was H + E).

## Discussion

In this largescale, singlecenter retrospective study spanning 8 years, we systematically characterized the epidemiological profile, clinical subtypes, diagnostic yield, and drug resistance patterns of EPTB among 2,281 patients. This study represents the first characterization of the epidemiological features of EPTB in Fuyang City, Anhui Province, China, along with an evaluation and analysis of diagnostic techniques. More importantly, using Omran’s classic epidemiological transition theory (14) as a heuristic analogy rather than a formal theoretical framework, we propose a ‘three-phase fluctuation’ model for EPTB to offer a tentative, hypothesis-generating interpretation of the dynamic changes in EPTB proportion observed over the study period. We acknowledge that the eight-year observation window is far shorter than the multi-decade societal shifts described by Omran, and that our observed fluctuations are largely attributable to changes in detection capacity and healthcare access rather than true incidence shifts. Thus, this framework should be viewed as an exploratory analytical lens, not a formal claim of epidemiological transition. Our findings highlight the substantial burden of EPTB, distinct demographic and clinical characteristics across subtypes, superior detection yield of IGRA (noting its inability to differentiate latent from active disease), and a notable proportion of MDR strains among resistant isolates.

We found that 28.46% of tuberculosis cases were diagnosed as EPTB. However, as this study was conducted at a single tertiary hospital, our cohort is likely enriched for severe or complicated cases, with underrepresentation of mild or communitymanaged cases. This referral bias may overestimate the proportion of EPTB (28.46%) relative to the general population, because complex extrapulmonary manifestations are more frequently referred to tertiary centers. This finding is lower than the 42.50% reported in 25 Egyptian governorates (15), the 51.90% reported in the Netherlands (16), and the 53.33% reported in Khuzestan Province, southwestern Iran (17), but higher than the 26.00% reported by 54 public health departments in Cologne, Germany (18), the 16.50% reported in India (19), the 7.00% reported in seven universityaffiliated tertiary hospitals in regions including Busan and Ulsan, South Korea (20). This marked geographical heterogeneity likely reflects the complex interplay of regional differences in host genetic susceptibility, HIV coinfection rates, prevalent *M. tuberculosis* genotypes, and the stringency of diagnostic algorithms for extrapulmonary disease, underscoring the critical influence of local epidemiological and healthcare system factors on TB clinical presentation.

The observed temporal fluctuations over the eightyear period—specifically the peak in 2020 (32.79%) followed by a gradual decline to the lowest level in 2025 (24.16%)—warrant careful interpretation beyond mere description. Omran’s epidemiological transition theory describes a threestage evolution of human disease patterns from infectious diseases to chronic diseases (14). The present study draws on this classic theory as an analytical framework, applying it creatively to extrapulmonary tuberculosis as a specific disease, and proposes a “three-phase fluctuation” hypothesis to explain the phased changes in the proportional composition of EPTB, offering a novel conceptual framework for EPTB surveillance in the postpandemic era.

Stage 1 (2018–2020): Diagnosisdriven Ascendancy. During this phase, the proportion of EPTB rose steadily from 29.07% to its apex of 32.79%, alongside an increase in absolute case numbers (from 273 to 296). This upward trend was paralleled by findings from central Guangxi, where EPTB proportions increased from 30.27% in 2016 to 35.93% in 2021, suggesting a broader regional pattern of increasing EPTB detection during this period (21). Consistent with this pattern, the ascendancy observed in our cohort was plausibly driven by the progressive rollout and clinical integration of highly sensitive immunological tools, particularly IGRA, in the Fuyang region. Enhanced detection of paucibacillary and subclinical EPTB forms likely shifted the notified case mix, artificially inflating the relative proportion of extrapulmonary disease before pulmonary case detection caught up. The concurrent rise in lymphatic TB during this period (from 4.22 to 7.33%) further supports this interpretation, as lymph node specimens—with their higher bacterial burden—would have been preferentially detected by the newly deployed molecular assays.

Stage 2 (2021–2022): Pandemicassociated Disruption. The proportion declined to 28.95%, accompanied by a reduction in absolute counts (from 296 to 270). This downturn is consistent with global evidence of healthcare system strain during the COVID19 pandemic. Globally, TB diagnoses fell by an estimated 21.0% in 2020 and 16.6% in 2021, with a 1.55 million decrease in diagnoses in 2020 alone (22). In the United States, reported TB diagnoses fell 20% in 2020 and remained 13% lower in 2021 than prepandemic levels (23). The WHO reported that TB notifications dropped 18% from 2019 to 2020 (from 7.1 to 5.8 million cases) (1). While hospitalized patients with complex EPTB continued to be captured, the overall reduction in case notifications suggests that milder EPTB presentations and routine PTB diagnoses were likely underdetected during periods of restricted healthcare access (24, 25). The sharp decline in “other TB” forms (from 7.83% in 2020 to 6.23% in 2023)—which include rare and often less severe presentations—provides additional circumstantial evidence for pandemicrelated underascertainment of nonemergent EPTB subtypes.

Stage 3 (2023–2025): Reequilibration and Proactive Control. The proportion fell further to a nadir of 24.16% by 2025. Notably, 2024 exhibited a marked surge in absolute case numbers (323 cases, the highest peak of the entire study period), which may represent a “catchup” effect of accumulated undiagnosed cases from the pandemic years. Globally, TB diagnoses rose from 7.5 million in 2022 to 8.2 million in 2023, and between 2023 and 2024, the global rate of people falling ill with TB declined by nearly 2% (1, 26). The 2024 peak in our cohort, occurring against a backdrop of declining global TB incidence, suggests that local catch-up detection rather than a true resurgence is the more plausible explanation. This interpretation is further supported by the subsequent sharp decline in 2025, which aligns with the restoration of routine TB services and enhanced active case-finding strategies that disproportionately improved pulmonary TB diagnosis, thereby recalibrating the EPTB/PTB ratio. The rebound of gastrointestinal TB from 1.56% in 2022 to 5.11% in 2025 may reflect restored access to endoscopic diagnostic services postpandemic, further supporting the “catchup” interpretation.

Taken together, this three-phase framework posits that while diagnostic advancements initially inflated EPTB proportions, the compounded effects of public health crises and subsequent proactive interventions may have fundamentally altered disease presentation and detection patterns. It is important to reiterate that these are observational trends, and causality cannot be definitively established from this retrospective design. It is important to reiterate that the phase breakpoints (2018–2020, 2021–2022, 2023–2025) are descriptive categorizations based on visual inspection of annual data, not statistically derived from formal change-point analysis. As such, they should be treated as exploratory and hypothesis-generating, and future studies with longer observation periods and consistent diagnostic algorithms are needed to validate or refine these temporal divisions. Longerterm, multicenter surveillance is required to validate this fluctuation framework and guide tailored TB control policies. Similar pandemicrelated shifts in TB case mix have been reported globally (22, 24, 25); however, our study is the first to explicitly articulate such a phased fluctuation framework, grounded in Omran’s classic epidemiological transition theory (14), specifically for EPTB in a Chinese prefecturelevel city.

Importantly, the three-phase framework does not imply a true change in EPTB incidence, but rather a change in the detection and reporting dynamics driven by external factors diagnostic technology rollout, healthcare access disruptions, and post-pandemic service restoration. This distinction is critical: the framework describes a fluctuation in the observed case mix, which may or may not reflect underlying disease incidence. Future studies incorporating population-based incidence data are needed to disentangle true epidemiological changes from detection artifacts.

The present study found that the majority of patients were aged ≥50 years (52.78%). This finding differs from the high incidence of EPTB reported in children under 15 years of age in Egypt (15) and the predominance of patients aged 20–29 years reported in Hunan, China (12). These discrepancies may be attributed to differences in geographical regions, population characteristics, and the epidemiological profiles of tuberculosis across studies. Interestingly, the bimodal age distribution observed in our cohort peaks in the 15–49 years (45.86%) and ≥50 years (52.78%) groups parallels the diagnostic shifts described in our threestage fluctuation framework. The younger peak likely reflects robust host immune responses that manifest as exudative forms such as pleural and lymphatic TB (which dominated the diagnosisdriven ascendancy stage), whereas the older age peak aligns with immunosenescence-related reactivation, manifesting as osteoarticular and CNS TB (27). This agesubtype interplay further underscores that epidemiological transitions are not merely statistical artifacts but are biologically and clinically grounded. Males accounted for 70.45% of cases, consistent with the wellrecognized male predominance in tuberculosis (12). Farmers comprised 70.50% of the cohort, highlighting substantial disease burden among agricultural workers, a pattern also observed in other regions of China and associated with ruralurban disparities in healthcare access and socioeconomic factors (28). A high burden of comorbidities was evident: pulmonary infection (73.74%) reflected that 54.45% of patients had concurrent pulmonary tuberculosis; hepatobiliary diseases (24.68%) and gastrointestinal diseases (20.34%) were prevalent, raising concern about druginduced hepatotoxicity during antituberculosis treatment, given that preexisting liver disease is a significant risk factor for antituberculosis druginduced liver injury (29); malnutrition (22.53%) was common, which may adversely affect treatment outcomes (30); and diabetes mellitus (9.30%) and hypertension (17.10%) were frequently observed, underscoring the need for integrated management of chronic diseases in EPTB care (31, 32). The high proportion of concurrent pulmonary tuberculosis (54.45%) emphasizes the potential for respiratory transmission and the importance of thorough pulmonary evaluation in all EPTB patients (12).

In the present study, pleural TB was the most common subtype (60.50%), followed by osteoarticular TB (12.98%) and lymphatic TB (6.97%). Consistently, pleural TB has been reported as the predominant EPTB subtype in multiple settings (8, 18, 19, 21, 33–35). This predominance likely reflects the ease of sampling pleural fluid and the high index of suspicion for tuberculosis pleurisy in endemic regions. The decline in osteoarticular TB from 19.92% in 2018 to 11.06% in 2025 suggests improved early diagnosis and treatment of primary TB, reducing hematogenous dissemination. The 2025 proportion (11.06%) closely aligns with the 11.3% reported in a US national study (36), indicating that proportional osteoarticular TB burden may stabilize at levels seen in lowincidence settings with sustained TB control efforts. The proportion of lymphatic TB in this study increased from 4.22% in 2018 to 10.64% in 2025, consistent with the notable resurgence of lymph node tuberculosis cases observed in Hunan Province during 2016–2017 (37), suggesting that the widespread adoption of diagnostic techniques and improved healthcare accessibility may have contributed to this upward trend. Notably, the contrasting trajectories of osteoarticular TB (declining) and lymphatic TB (rising) may reflect differential impacts of the threestage transition on subtypes with distinct pathophysiological mechanisms: osteoarticular TB, typically resulting from hematogenous dissemination of primary infection, would be expected to decline with improved early diagnosis and treatment of pulmonary TB, whereas lymphatic TB—often presenting with accessible specimens amenable to molecular diagnosis—would be more readily captured during the diagnosisdriven ascendancy stage.

Consistent with the findings by Pang et al., who reported a culture positivity rate of 12.8% (758/5,910) for EPTB (33), the present study demonstrated a similarly low yield of mycobacterial culture (14.53%, 119/819), reflecting the paucibacillary nature of EPTB specimens (38, 39). In contrast, IGRA demonstrated a high positivity rate (88.04%). However, IGRA detects immune response, not active disease, and cannot distinguish latent from active tuberculosis (40, 41). Therefore, while IGRA may serve as a useful adjunctive tool, its results should be interpreted with caution and not overinterpreted as a diagnostic gold standard. A proper evaluation requires consideration of both sensitivity and specificity, not merely positivity rates. GeneXpert MTB/RIF Ultra showed a moderate positivity rate (35.60%), which aligns with its improved sensitivity over conventional molecular methods but remains limited by the low bacterial load in extrapulmonary samples (42). These findings underscore the complementary roles of immunological and molecular assays in enhancing the detection sensitivity for EPTB. EPTB is often paucibacillary and exhibits uneven bacterial distribution, which constrains traditional microbiological methods (38). Although Xpert Ultra incorporates multicopy targets and achieves a lower limit of detection, its sensitivity remains suboptimal in smearnegative or extrapulmonary specimens (42). IGRA detects IFNγ release by T cells in response to MTBspecific antigens and is not directly limited by lesion site or bacterial load (40). However, despite its high sensitivity, IGRA cannot distinguish latent from active tuberculosis, and its specificity may decline in highburden settings (41). Thus, IGRA offers a high positivity rate but cannot confirm active disease, and its specificity cannot be assessed without a reference standard. GeneXpert MTB/RIF Ultra, while less sensitive in our cohort (based on positivity rate), provides concurrent rifampicin resistance information for therapeutic guidance. Specifically, detection of rifampicin resistance enables clinicians to avoid ineffective rifampincontaining regimens and promptly initiate alternative treatments (e.g., fluoroquinolonebased or secondline drug therapies), which is critical for improving patient outcomes and preventing transmission of drugresistant strains. An integrated approach that combines clinical context, site of involvement, and complementary tests is essential for accurate diagnosis. Such integration ensures that the limitations of individual tests such as IGRA’s inability to distinguish latent from active disease or Xpert Ultra’s reduced detection in paucibacillary specimens are not interpreted in isolation, thereby guiding appropriate treatment decisions (e.g., when to treat empirically, when to repeat testing, or when to use alternative regimens based on resistance profile) and avoiding unnecessary or incorrect therapy (43).

Importantly, a direct comparison of IGRA, Xpert, and culture without considering specimen type and anatomic site is not fully fair or clinically contextualized. Recent reviews have highlighted that IGRA, as a bloodbased test measuring IFNγ release from sensitized T cells, is unaffected by BCG vaccination but cannot distinguish latent from active tuberculosis, and its diagnostic utility varies by disease site and host immune status (44). In the present study, diagnostic yields differed substantially when stratified by specimen type and anatomic site. For pleural fluid (the most common specimen, accounting for 49.58% of culturepositive cases), IGRA positivity was high (>85% in pleural TB) but could not differentiate active pleural disease from latent infection; Xpert Ultra yielded a moderate positivity rate (25.91% in pleural TB), and culture positivity was low (10.95%). For tissue biopsy specimens (e.g., lymph node tissue), both Xpert Ultra (80.00% in lymphatic TB) and culture (20.45%) achieved higher yields due to the higher bacterial burden in solid tissue samples. For pus aspirates (e.g., from cold abscesses in osteoarticular TB), Xpert Ultra and culture showed moderate to high positivity (51.02 and 36.25%, respectively). For cerebrospinal fluid (CSF) in central nervous system (CNS) TB, all three methods performed poorly: culture positivity was extremely low (2.44%), Xpert Ultra was 29.09%, and IGRA on blood has limited diagnostic value for CNS TB (45).

The sensitivity of molecular and immunological tests varies markedly with sample type here we refer to detection yields, not formal sensitivity. Recent studies have similarly emphasized that the sensitivity of molecular and immunological tests varies markedly with sample type, and that an integrated approach using multiple modalities is essential for EPTB diagnosis (43, 45).

In this study, significant heterogeneity was observed across the five most common singlesystem EPTB subtypes in terms of demographics, comorbidities, and diagnostic test performance. When further stratified by specimen type (fluid, tissue, CSF, pus), the following patterns emerged: (1) pleural fluid (pleural TB)—IGRA high but nonspecific, Xpert moderate, culture low; (2) lymph node tissue (lymphatic TB)—Xpert highest (80.00%), culture moderate (20.45%), IGRA very high (96.36%) but unable to confirm active infection; (3) synovial fluid/pus (osteoarticular TB)—Xpert (51.02%) and culture (36.25%) both useful; (4) CSF (CNS TB)—all methods suboptimal, with culture only 2.44%. Pleural TB predominated in males, osteoarticular TB mainly affected older males, lymphatic TB was more common in younger adults with nearequal sex distribution, and CNS and gastrointestinal TB also predominantly affected males and older adults; the high proportion of rural residents across subtypes highlights the role of socioeconomic and occupational factors in disease susceptibility, findings consistent with previous studies (8, 33). Comorbidity profiles varied substantially, with concurrent pulmonary TB, hepatobiliary disease, and malnutrition being most prevalent in CNS and gastrointestinal TB, HIV/AIDS also most prevalent in CNS and gastrointestinal TB, consistent with immunosuppressionassociated dissemination (27), and diabetes more frequent in osteoarticular and CNS TB. As noted above, diagnostic performance also differed markedly by specimen type and site: IGRA positivity was highest in lymphatic TB (96.36%), Xpert MTB/RIF Ultra achieved the highest positivity rates in lymph node (80.00%) and osteoarticular TB (51.02%), consistent with the higher bacterial burden in these specimens, while MTB culture positivity was highest in osteoarticular TB (36.25%) but remained extremely low in CNS TB (2.44%) and gastrointestinal TB (6.67%), underscoring the limitations of traditional microbiological methods for these forms; the low yield in CNS and gastrointestinal TB underscores the critical need for more sensitive molecular platforms, such as Xpert MTB/RIF Ultra or targeted nextgeneration sequencing, for paucibacillary specimens (43). These findings support the need for subtypespecific diagnostic algorithms and comprehensive comorbidity management to improve detection yield and clinical outcomes in EPTB.

Among culturepositive EPTB cases, pleural fluid accounted for nearly half (49.58%), followed by pus (19.33%) and biopsied tissue (10.92%). This distribution is consistent with findings from Saudi Arabia, where pleural fluid constituted 21.18% of EPTB specimens submitted for culture testing, while tissue and pus accounted for 44.62 and 9.82%, respectively (46). However, the higher proportion of culturepositive pleural fluid in our cohort may reflect the higher burden of pleural tuberculosis in the study population, as well as differences in clinical sampling practices. These findings suggest that fluid and tissue specimens from accessible sites yield higher culture positivity, whereas cerebrospinal fluid and ascitic fluid have limited diagnostic utility. This is partly attributed to the paucibacillary nature of EPTB, which reduces the sensitivity of microbiological detection methods, including culture, PCR, and microscopy (39). For suspected CNS TB, given the extremely low culture yield, molecular assays such as Xpert Ultra or nextgeneration sequencing should be prioritized over culture alone (47).

In the present study, drug susceptibility testing (DST) was performed on only 54 *Mycobacterium tuberculosis* isolates from culturepositive EPTB cases. This sample size is relatively small compared with the total cohort, and these isolates may not be representative of all EPTB cases. Consequently, the resistance rates reported here may underestimate the true burden of drug resistance, and findings should be interpreted with caution. The overall resistance rate among these 54 isolates was 16.67%, with MDRTB accounting for 9.26% of all isolates and 55.6% of resistant strains. INH resistance was the most common firstline drug resistance (14.81%), which is generally consistent with national surveillance data indicating INH as the predominant resistance pattern in China (48). Notably, RFP resistance was exclusively observed in MDR isolates, a finding that contrasts with spinal TB studies in southcentral China where the Beijing genotype—strongly associated with MDR—predominated in retreatment cases with higher resistance rates (49). These comparisons suggest that while INH remains the most frequent resistance among firstline drugs, resistance profiles may vary by geographic region and specimen type. However, given the limited DST sample size, no strong conclusions about resistance patterns can be drawn. Nonetheless, the emergence of MDREPTB highlights the importance of routine drug susceptibility testing to guide appropriate treatment, and larger, more representative studies are needed to better characterize resistance in EPTB. From the perspective of our threestage fluctuation framework, the stable MDR proportion (9.26% of all tested isolates) across the study period suggests that drug resistance patterns are less susceptible to shortterm epidemiological transitions than case detection dynamics, reinforcing the need for sustained, uninterrupted DST capacity even as healthcare systems undergo transitional phases.

## Conclusion

This largescale, singlecenter retrospective study provides the first comprehensive characterization of EPTB in Fuyang City, Anhui Province, China, and introduces, using Omran’s classic epidemiological transition theory (14) as a heuristic analogy, a ‘three-phase fluctuation’ framework to offer a tentative interpretive lens for the dynamic changes in EPTB proportion over an eight-year span. This framework is descriptive and hypothesis-generating, and its phase breakpoints are based on annual trend observations rather than statistical validation. We demonstrate that the EPTB proportion has transitioned from a diagnosisdriven peak (Stage 1, 2018–2020), through pandemic disruption (Stage 2, 2021–2022), to a postpandemic reequilibration phase (Stage 3, 2023–2025). Our findings reveal that EPTB accounts for a substantial proportion of tuberculosis cases, with distinct demographic and clinical features across subtypes, and highlight significant geographical heterogeneity in EPTB epidemiology. Pleural TB remains the predominant subtype, while temporal trends show a decline in osteoarticular TB and a rise in lymphatic TB, suggesting evolving epidemiological dynamics. The diagnostic evaluation demonstrates that IGRA offers the highest positivity rate (detection yield) among the assays studied, whereas GeneXpert MTB/RIF Ultra provides moderate positivity with the added value of rifampicin resistance detection, and culture yields remain low due to the paucibacillary nature of EPTB. It is important to note that IGRA cannot distinguish latent from active tuberculosis, and therefore its results should always be interpreted in the context of clinical findings and other microbiological tests. The notable proportion of multidrug-resistant strains among resistant isolates underscores the urgent need for routine drug susceptibility testing to guide appropriate treatment. The identification of MDR strains among the resistant isolates in this selected cohort highlights the need for routine DST, although the generalizability of these findings requires confirmation in larger, unselected populations. Subtypespecific differences in demographics, comorbidities, and diagnostic yields further emphasize the importance of tailored diagnostic algorithms and integrated comorbidity management. The three-phase model proposed herein not only applies Omran’s classic epidemiological transition theory (14) creatively to extrapulmonary tuberculosis as a specific disease, contextualizing our empirical observations within a theoretical framework, but also offers a transferable analytical lens for other highburden regions seeking to understand postCOVID19 TB epidemiology. This study contributes valuable baseline data for EPTB surveillance in Anhui Province and highlights the critical need for enhanced diagnostic strategies, subtypespecific clinical approaches, and sustained efforts to monitor drug resistance in the context of EPTB control.

### Limitations

This study has several limitations. First, as a singlecenter retrospective analysis, the findings may not be generalizable to other regions in China. Second, reliance on hospital records may introduce selection bias, as our cohort likely represents more severe or complex cases referred to a tertiary center. Third, changes in diagnostic practices and reporting standards over the eightyear period could have influenced the observed temporal trends and should be considered as alternative explanations for the observed fluctuations, rather than definitive proof of the proposed three-phase framework. Fourth, data on key variables such as specific symptom duration, treatment outcomes, and socioeconomic status were not available for analysis. Fifth, the sample sizes for certain EPTB subtypes (e.g., CNS and gastrointestinal TB) were relatively small, limiting the statistical power for subgroup analyses. Sixth, the IGRA results reflect tests performed at a single time point and cannot distinguish between latent infection and active disease, potentially overestimating its diagnostic specificity. Seventh, as the three-phase fluctuation framework is proposed primarily based on temporal correlations rather than causal evidence, and its phase breakpoints are descriptively rather than statistically derived, it should be viewed as a hypothesis-generating framework requiring validation through multicenter, longer-term studies with consistent diagnostic algorithms and formal change-point analyses. Eighth, pyrazinamide (PZA), a standard first-line anti-TB agent, was not included in the DST panel; consequently, the term ‘first-line drug resistance’ in our analysis refers exclusively to the tested agents (isoniazid, rifampicin, and ethambutol) and does not account for PZA resistance, which may lead to an underestimation of overall first-line drug resistance in this cohort. Finally, we did not perform genomic sequencing to characterize resistance mutations, which could have provided additional insights into the molecular epidemiology of drug-resistant EPTB.

## Data Availability

The original contributions presented in the study are included in the article/supplementary material, further inquiries can be directed to the corresponding author/s.
